# Supplementary material for: Clinical outcomes in patients with atrial fibrillation treated with DOACs in a specialized anticoagulation center: Critical appraisal of real-world data
Source: PLoS One. 2023 Feb 24;18(2):e0279297. doi: 10.1371/journal.pone.0279297 (PMC9955586; doi:10.1371/journal.pone.0279297)
Supplement: S1 File — (PDF) [file pone.0279297.s002.pdf]

S1 Table. Descriptive data for the patients with Edoxaban, stratified by age and by sex

	All	≤ 74 years	75-84 years	≥ 85 years	Men	Women
**N**	**615**	195	288	132	316	299 ‡
**Age**; mean (SD)	78 (7.8)	68.9 (5.0)	79.6 (2.8) ‡	87.8 (2.4) ‡	76.8 (8.2)	79.2 (7.2) ‡
**Male sex**; N (%)	316 (51.4)	112 (57.4)	145 (50.5)	58 (43.9) †	316 (100)	0 (0)
**Naive**; N (%)	80 (13)	29 (14.9)	35 (12.2)	16 (12.1)	44 (13.9)	36 (12.0)
**CHA_2DS_2-VASc**; mean (SD)	3.97 (1.52)	3.03 (1.44)	4.29 (1.33) ‡	4.65 (1.33) ‡	3.55 (1.50)	4.40 (1.41) ‡
**HAS-BLED**; mean (SD)	2.1 (0.76)	1.95 (0.84)	2.15 (0.70) ‡	2.21 (0.74) ‡	2.18 (0.83)	2.02 (0.68) †
**CCI***; mean (SD)	1.46 (1.44)	1.45 (1.56)	1.42 (1.41)	1.56 (1.29)	1.73 (1.62)	1.17 (1.14) ‡
**Hemoglobin;** mean (SD), g/L	133 (17)	137 (18)	132 (16) †	131 (14) ‡	137.8 (18)	128.3 (13.4) ‡
**Bilirubin**; mean (SD)	12.8 (5.9)	11.8 (5.0)	13.1 (5.8)	13.9 (7.4)	14.1 (6.5)	11.6 (5.0) ‡
**AST**; mean (SD)	21.1 (8.1)	21.4 (7.9)	20.8 (8.5)	21.0 (7.4)	21.2 (8.7)	20.9 (7.4)
**ALT**; mean (SD)	18.8 (11.0)	21.9 (12.2)	17.9 (10.8) ‡	16.3 (8.3) ‡	19.6 (11.6)	18.0 (10.2)
**CrCl****; mean (SD), mL/min	62.9 (22.5)	77.2 (23.5)	60.1 (19.7) ‡	47.9 (12.9) ‡	67.4 (24.7)	58.1 (18.9) ‡
**CrCl** < 30 mL/min**; N (%)	13 (2.1)	0 (0)	5 (1.7)	8 (6.1) ‡	1 (0.3)	12 (4.0) ‡
**BMI**; mean (SD), kg/m^2	27.02 (4.87)	27.98 (5.49)	27.01 (4.80)	25.64 (3.60) ‡	27.23 (4.47)	26.80 (5.26)
**Antiplatelets**; N (%)	42 (6.8)	21 (10.8)	16 (5.6) †	5 (3.8) †	34 (10.8)	8 (2.7) ‡
**Previous stroke**; N (%)	72 (11.7)	17 (8.7)	31 (10.8)	24 (18.2) †	42 (13.3)	30 (10.0)
**Previous major bleeding**; N (%)	73 (11.7)	26 (13.3)	34 (11.8)	13 (9.8)	44 (13.9)	29 (9.7)

*CCI: Charlson Comorbidity Index

** CrCl: creatinine clearance from Cockcroft-Gault formula (mL/min).

† Statistically significant vs reference group (≤ 74 years for age and men for sex) with P<0.05.

‡ Statistically significant vs reference group (≤ 74 years for age and men for sex) with P<0.01.

S2 Table. Incidence of complications with edoxaban, by age and sex

	All	≤ 74 years	75-84 years	≥ 85 years	Women	Men
N	615	195	288	132	299	316
Total follow-up (years)	801.62	237.23	386.97	177.42	406.64	394.98
Follow-up (months); med [P_{25}-P_{75}]	15.0 [7.9-24.4]	13.0 [7.3-23.2]	15.8 [8.6-24.6]	15.7 [7.4-25.0]	16.3 [8.3-24.9]	13.9 [7.5-23.8]
Age (years); mean (SD)	78 (7.8)	68.9 (5.0)	79.6 (2.8)	87.8 (2.4)	79.2 (7.2)	76.8 (8.2)
**Major thrombotic complications (stroke/systemic embolism)**						
CHA_2DS_2-VASc; mean (SD)	3.97 (1.52)	3.03 (1.44)	4.29 (1.33)	4.65 (1.33)	4.40 (1.41)	3.55 (1.50)
Number of events	8	4	2	2	2	6
Incidence rate; % py (95%CI)	1.01 [0.43-1.98]	1.69 [0.46-4.37]	0.52 [0.06-1.88]	1.14 [0.14-3.12]	0.49 [0.06-1.77]	1.52 [0.56-3.31]
**Major hemorrhagic complications**						
HAS-BLED; mean (SD)	2.1 (0.76)	1.95 (0.84)	2.15 (0.70)	2.21 (0.74)	2.02 (0.68)	2.18 (0.83)
Number of events	28	4	15	9	13	15
Incidence rate; % py (95%CI)	3.52 [2.34-5.09]	1.69 [0.46-4.37]	3.91 [2.19-6.45]	5.13 [2.34-9.73] †	3.20 [1.70-5.47]	3.80 [2.13-6.26]
**Composite (major thrombotic and hemorrhagic complications)**						
Number of events	36	8	17	11	15	21
Incidence rate; % py (95%CI)	4.53 [3.17-6.27]	3.39 [1.46-6.67]	4.43 [2.58-7.10]	6.27 [3.13-11.21]	3.70 [2.07-6.08]	5.32 [3.29-8.13]
**Clinically relevant non-major thrombosis**						
Number of events	8	0	6	2	5	3
Incidence rate; % py (95%CI)	1.01 (0.43-1.98)	0.00 [0.00-1.56]	1.56 [0.57-3.40]	1.14 [0.14-4.12]	1.23 [0.40-2.87]	0.76 [0.16-2.22]
**Clinically relevant non-major bleeding**						
Number of events	64	19	30	15	22	42
Incidence rate; % py (95%CI)	8.05 [6.20-10.27]	8.04 [4.84-12.56]	7.82 [5.28-11.16]	8.55 [4.78-14.09]	5.41 [3.39-8.19]	10.63 [7.66-14.37]
**All-cause mortality**						
Number of events	40	7	13	20	21	20
Incidence rate; % py (95%CI)	5.03 [3.59-6.85]	2.96 [1.19-6.11]	3.39 [1.80-5.79]	11.39 [6.96-17.60] ‡	5.16 [3.20-7.89]	5.06 [3.09-7.82]

% py = per 100 patient-years

† Statistically significant vs ≤ 74 year-old group with $P<0.05$.

‡ Statistically significant vs ≤ 74 year-old group with $P<0.01$.

S3 Table. Incidence of complications in naive patients

	All	Edoxaban	Apixaban	Dabigatran	Rivaroxaban
N	185	90	52	37	6
Total follow-up (years)	166.51	77.7	45.83	38.46	4.52
Follow-up (months); med [P_{25}-P_{75}]	10.1 [6.6-14.1]	8.9 [6.1-14.1]	10.3 [6.3-14.5]	10.4 [7.4-14.8]	8.6 [2.1-13.5]
**Major thrombotic complications (stroke/systemic embolism)**					
Number of events	4	1	1	2	0
Incidence (95%CI)	2.40 (0.66 – 6.15)	1.29 (0.03-7.17)	2.18 (0.06-12.16)	5.2 (0.63-18.79)	0.00 (0.00-81.51)
**Major hemorrhagic complications**					
Number of events	5	3	1	1	0
Incidence rate; % py (95%CI)	3.00 (0.98 – 7.01)	3.86 (0.80-11.28)	2.18 (0.06-12.16)	2.60 (0.07-14.49)	0.00 (0.00-81.51)
**Composite (major thrombotic and hemorrhagic complications)**					
Number of events	9	4	2	3	0
Incidence rate; % py (95%CI)	5.41 (2.47 – 10.26)	5.15 (1.40-13.18)	4.36 (0.53-15.76)	7.80 (1.61-22.80)	0.00 (0.00-81.51)
**Clinically relevant non-major thrombosis**					
Number of events	2	2	0	0	0
Incidence rate; % py (95%CI)	1.20 (0.15 – 4.34)	2.57 (0.21.9.30)	0.00 (0.00-8.05)	0.00 (0.00-9.59)	0.00 (0.00-81.51)
**Clinically relevant non-major bleeding**					
Number of events	11	5	2	4	0
Incidence rate; % py (95%CI)	6.61 (3.30 – 11.82)	6.44 (2.09-15.02)	4.36 (0.53-15.76)	10.40 (2.83-26.63)	0.00 (0.00-81.51)
**All-cause mortality**					
Number of events	14	7	4	2	1
Incidence rate; % py (95%CI)	8.41 (4.60 – 14.11)	9.01 (3.62-18.56)	8.73 (2.38-22.35)	5.20 (0.63-18.79)	22.12 (0.56-123.27)

% py = per 100 patient-years

† Statistically significant vs edoxaban group with P<0.05.

‡ Statistically significant vs edoxaban group with P<0.01.
